# A Survey of Work-Related Pain Prevalence Among Construction Workers in Hong Kong: A Case-Control Study

**DOI:** 10.3390/ijerph16081404

**Published:** 2019-04-18

**Authors:** Joanne W.Y. Chung, Henry C.F. So, Vincent C.M. Yan, Phoebe S.T. Kwok, Bonny Y.M. Wong, Jackie Y. Yang, Albert P.C. Chan

**Affiliations:** 1 Department of Health and Physical Education, The Education University of Hong Kong, 10 Lo Ping Road, Tai Po, New Territories, Hong Kong; cmyan@eduhk.hk (V.C.M.Y.); stkwok@eduhk.hk (P.S.T.K.); bymwong@eduhk.hk (B.Y.M.W.); 2 School of Nursing, Guangzhou Medical University, 195 Dongfeng Xi Road, Guangzhou 510182, China; 3 Department of Mathematics and Information Technology, The Education University of Hong Kong, 10 Lo Ping Road, Tai Po, New Territories, Hong Kong; hcfso@eduhk.hk; 4 Department of Building and Real Estate, The Hong Kong Polytechnic University, Hunghom, Kowloon, Hong Kong; jackie.yyang@polyu.edu.hk (J.Y.Y.); albert.chan@polyu.edu.hk (A.P.C.C.)

**Keywords:** pain prevalence, musculoskeletal pain, construction worker, case-control study

## Abstract

Construction workers undertake demanding physical work and face high risk of injuries in poor working environments. This case-control study investigated the extent of their musculoskeletal pain incidence at work. A total of 2021 construction workers in different trades were interviewed on-site in a survey from December 2017 to December 2018. The survey results revealed that the pain prevalence of the subjects in the last 24 h was 10.6 %. The worst and top most common pain spots caused by work were central lower back, left/right shoulders, and knees. Regarding pain management, their most common method was to ignore the pain (21.4%). The average percentage of pain relief after receiving treatment in the 24 h was 37.12%. Besides, significant differences were found between the pain and non-pain groups regarding their employment duration in current job or their average sleep duration in the 24 h. The study showed that those with multiple and bilateral pain sites had pain interference on their living activities.

## 1. Introduction

Construction workers spend most of their working hours at the workplace. Their workplace environment is related directly to their health and well-being [1]. Construction sites are generally known to be one of the workplaces with high risk of injuries and poor health [2,3,4,5,6]. Construction workers, in particular, are prone to painful musculoskeletal strains and injuries because of their labourious and strenuous manual jobs [7]. The pain severity is aggravated by their harsh working environment, which is associated with irregular working hours, unstructured mealtimes, tough and dusty work, and outdoor and indoor heat, etc. [8].

Pain prevalence has been studied in the local community for decades and is useful in the evaluation of the extent of people’s pain suffering and its burden on the healthcare system. Even though construction workers have been found to suffer from musculoskeletal pain, local attention given to this group is comparatively little. Overseas studies showed different prevalence rates as a result of using different periods and case definitions, but musculoskeletal pain which was characterised by multiple pain sites was consistent in most construction trades [9,10,11,12]. In Saudi Arabi, for example, it was reported that all their subjects (*n* = 165) had reported pain in the upper and lower limbs, backs, and knees [9]. Though the study had a small sample size and provided unclear information on multiple pain sites per subject, it did highlight the significance of pain as a health challenge to the construction industry.

In a systematic review of musculoskeletal symptoms in the construction industry, it was reported that the range of one-year pain prevalence rate was from 15.1% for hip/thigh to 51.1% for low back [13]. It is important to note the point prevalence as well because it refers to the pain which is the actual ongoing suffering of the workers concerned at the time of reporting. In the cited systematic review, the point prevalence, though heterogeneity was noted, for neck was 5.5–22.0%, shoulder 10.5 to 28.7%, elbow 12.0%, wrist/hand 21.0–28.4%, upper back 6.2–14.0%, lumbar 16.5–60.3%, hip/thigh 11.0%, knee 22.0%, and ankle/foot 13.4–19.0%. The various studies in the review were done in between 1996 and 2013. The team would argue that the changes in technology and facilities since then might have changed these figures. Furthermore, most of the studies just reported pain prevalence and very rarely took into account the severity of pain and its impact on living activities and pain relief methods [14]. In two other studies, the risk factors that had caused pain were reported to be job-required repetitive lifting [15] and fatigue [16]. Furthermore, it was reported in another study that job-related stresses together with age, physical workload, effort-reward, job strain, and organizational injustices were all factors associated with musculoskeletal pain [17]. These causes could also be applicable to the construction industry [18].

Urban Asia, especially Hong Kong, is undergoing a construction boom today and yet we have limited understanding of the pain prevalence among our construction workers. In Hong Kong, there were about 480,998 registered construction workers in which the female to male ratio was 9.65 % to 90.35 % in 2019 [19].

In the construction industry, it is common to find people still at work as long as they can tolerate the pain. It has been found in a 14-year prospective study that people with multiple pain sites on their body continued to report multiple pain sites at the end of the 14-year period [20]. To avoid prolonged pain suffering in later life, it is advisable to identify pain even when it is less than intolerable and to intervene as soon as possible. In this connection, it is critical to study point prevalence that can inform the stakeholders the proportion of people who are in pain but still at work.

This study is part of a health-profiling initiative supported by the Construction Industry Council (CIC) of Hong Kong. The objectives of this initiative are to provide descriptive statistics of the demographic characteristics and comprehensive health profile of the local construction workers. After the health problems have been identified, measures can be designed and implemented for improving the physical and psychological health of the workers. This study aimed at determining the pain prevalence, reviewing the extent of the problem and workers’ pain relief behaviours, and identifying the associated risk factors for pain. The study attempted to answer the following research questions:

- What was the point prevalence of pain?
- What was the pain severity?
- What was the pain interference?
- What were their pain relief behaviours?

## 2. Materials and Methods

This was a case-control study held between December 2017 and December 2018 in a health-profiling initiative supported by the CIC (Grant Number: K-ZB93). Phase 1 of the initiative was held from July 2014 to August 2016. About 1200 workers’ health profiles have been recorded. The data of this study was from Phase 2 which was started at December 2017.

The research team had visited the construction sites all around Hong Kong. Research personnel stayed in the sites from 10:30 to 13:30 and recruited the construction workers by convenience sampling during their lunch breaks. All registered construction workers were invited to join the study. A total of 2021 construction workers in different construction trades were recruited. Considering the total number of registered construction worker in Hong Kong, with 5% margin of error and 95% confidence level, the estimated sample size required was 384 per group (https://www.checkmarket.com/sample-size-calculator/).

After obtaining their written consent, their body height, body weight and peak expiratory flow rate (measured by peak flow meter) were measured; then an electronic form questionnaire (e-questionnaire) was administered to the workers by the trained student helpers and their answers were inputted into a tablet computer. All the data were sent to a well-secured server through mobile network immediately. The e-questionnaire covered the information on the workers’ demographics, job categories, work experience, dietary habit, warm-up and stretching exercises, sleep, recreation, and habits of drinking and smoking and health literacy (measured by the European Health Literacy Questionnaire (Q16)). Items in the e-questionnaire were basically extracted from the population health survey conducted by the local governmental health authority [21].

The pain data is part of the health profiling initiative and they were collected by the Brief Pain Inventory (BPI) with established structural validity and internal consistency [22,23]. There were nine questions covering pain intensity, pain sites, pain relief methods, and pain interference. For the question on pain sites, the workers were asked to shade the area(s) where they felt pain in a human full body diagram with front and back views, and they also required to put an “X” on the area that hurt most. The BPI took about 5 min for a worker to complete.

As there were more than 20 construction trades, we have grouped them into three categories according to their intensity of activities for data analysis. The intensity of activities were expressed as the ratio of work metabolic rate to resting metabolic rate (METs) and the three work intensity categories are light (METs < 3.0 kcal/kg/h), moderate (METs 3.0–6.0 kcal/kg/h) and heavy (MET > 6.0 kcal/kg/h) [24,25].

The study was approved by The Education University of Hong Kong (EdUHK) and ethics approval was obtained prior to the commencement of the study (Human Research Ethics Committee Reference Number: 2017-2018-0259).

The demographic characteristics, trades distribution, behavioural characteristics, pain intensities and interference scores of the construction workers were reported using descriptive statistics. The comparisons between pain and non-pain groups were analyzed by chi-square (X^2^) for frequency counts. For pain scores, the comparisons between gender and age groups were done by independent sample *t*-test and analysis of variance (ANOVA). All results were considered as statistically significant when *p*-value was smaller than 0.05.

## 3. Results

Among the 2021 subjects, 356 (17.6%) were female and 1665 (82.4%) male. There were no significant differences in gender, age group, ethnicity, education level, and work intensity between the pain and non-pain groups (Table 1).

A total of more than 20 construction trades were included in the sample (Table 2). About 40% of them were general labourers. Table 3 shows the behavioural characteristics of all workers by pain status. The trades were grouped according to work intensity (light, moderate, and heavy) for analysis [24,25]. A majority of them had moderate work intensity. About 60% of them were on daily wages, without job security or any long-term employment benefits.

There were significant differences in pain status for their years of service in current companies (df = 252.314, *t* = −2.122, *p* = 0.035) and average daily sleep hours (df = 256.343, *t* = 2.804, *p* = 0.005). Specifically, those with pain had stayed with the same company for a longer period of time and those slept less hours suffered more pain. After further statistical analysis, there was significant correlation between pain and average sleep hours (*r* = −0.066, *p* = 0.003). However, there were no associations of pain with gender, work intensity, age, or body mass index.

### 3.1. Characteristics of Pain and Pain Management

#### 3.1.1. Pain Prevalence and Pain Intensity

The pain prevalence in the last 24 h was found to be 10.6 % (215/2021). As for pain intensity, it was measured on a 0–10 scale, with 0 as ‘no pain’ and 10 as ‘pain as bad as it could be’. Table 4 shows the pain intensities (worst, least, average, and current) as reported by the workers and the level of pain was mild [26].

#### 3.1.2. Pain Location and Pain Sites

**Figure 1:**
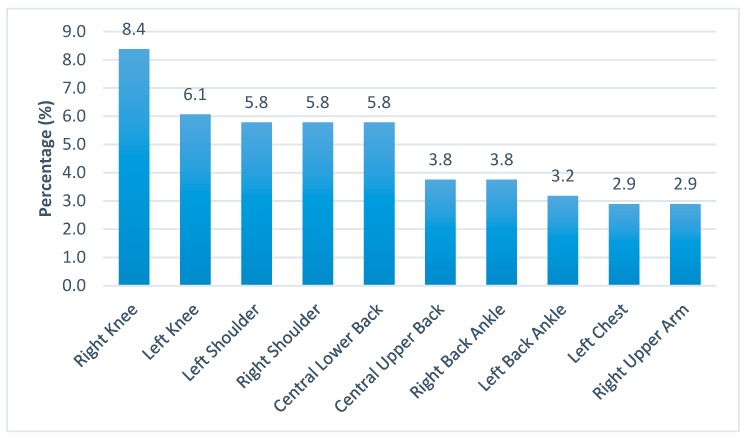
Percentage distribution of the top 10 common pain sites.

**Figure 2:**
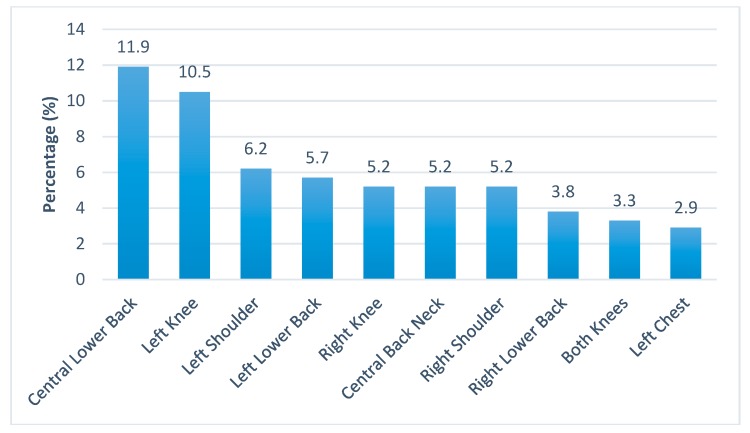
Percentage distribution of top 10 worst pain sites.

As indicated by the subjects, the average number of pain sites per subject was 2.58 (Standard Deviation, SD = 1.54). The first five common pain sites were right and left knees, left and right shoulders, and the central lower back (Figure 1). The first two worst pain sites were central lower back and left knee, while the rest of the sites were mostly on the four limbs (Figure 2).

#### 3.1.3. Pain Interference

The most affected domains of living activities were work, daily life, and sleep (Table 5). There was no significant gender difference in pain interference.

The BPI has two main scores which are the pain severity score (0–40) and the pain interference score (0–70). The total score ranges from 0 to 110. The workers’ pain intensity scores were calculated based on the BPI for subsequent analysis (Table 6). There were no significant differences with respect to gender in their reported pain severity, pain interference score, and total pain score (Table 7). It was noted that males reported less pain severity, pain interference, and total pain. Whilst there were also no statistically significant differences on all pain scores between age groups (Table 8).

For subjects with multiple pain sites (i.e., the total number of pain site is >1), significant differences were found on the pain interference score (Mean difference = 4.50, *t* = −2.188, *p* = 0.030) and the total pain score (Mean difference = 5.34, *t* = −2.119, *p* = 0.035) between those with and without multiple pain sites (Table 9). 

As regards symmetrical pain sites (i.e., pain sites present in both the left and right side of the body, including anterior and posterior sides of torso, limbs, and soles, e.g., left and right shoulder), significant differences were also found on the pain interference score (Mean difference = 5.89, *t* = −2.934, *p* = 0.004) and the total pain score (Mean difference = 7.39, *t* = −3.003, *p* = 0.003) between the subjects with and without bilateral pain sites. However, there were no statistically significant differences on all the pain scores with respect to the subjects having or not having warm-up and cool-down exercises before and after work respectively (Table 10).

#### 3.1.4. Pain Relief Methods

**Figure 3:**
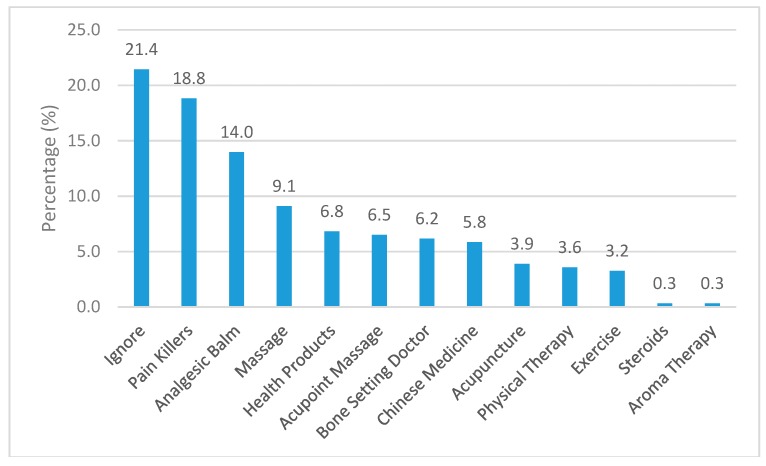
Percentage distribution of pain relief methods used.

With regard to the pain relief methods, the subjects indicated that about 21% of the time they chose to ignore the pain and this was the most frequent method used. Two other frequent methods used were taking pain killers (18.8%) and applying analgesic balm (14.0%). Other less frequent methods included massage, acupoint massage, Chinese medicine, and so on. Only 3.2% of the time they used exercises as a means for pain relief (Figure 3). About 37.21% of them indicated that pain was relieved (80/215). There was no significant difference with respect to gender in choosing pain relief methods.

## 4. Discussion

The pain prevalence revealed in this study was consistent with that of those reported in a recent systematic review [13]. Since this study was on point prevalence, i.e., pain in the last 24 h, it was reasonable to have workers reporting mild pain because those who had moderate or severe pain might probably not be at work. The study suggested that males had higher pain tolerance than females which was consistent with other research findings [27]. This might have resulted in a lower reported pain intensity in male workers. However, one must take note that this does not necessarily mean the cell damages are lesser.

The first five common pain sites are the weight bearing areas of the body. The most common site was the right knee and most of the people are right-handed. For the right-handed subjects, they tended to shift their weight onto their left knees to reduce any discomfort or pain on the right (compensatory adjustments in postural control) [28]. Thus, it was reasonable to find the second common pain site was the left knee. Shoulder pain was most likely to be due to imbalanced muscle strength caused by heavy lifting or manual work that required repetitive physical exertion of the upper limbs. Central lower back was the centre of the core trunk which was the pivot to provide an anchor for the four limbs. Again, any imbalance in muscle strength will cause back pain which further weakens the core stability of the workers [29] and increases their risks of injuries and falls.

The first few worst pain sites as reported by the workers were the left weight bearing joints and the central lower back despite the right knee was the most common pain site. The team argued that this might be due to the pain in the right knee of the workers, being the most common pain site and as a compensatory mechanism, had to be replaced by the use of the left side of their body and core muscle to perform their jobs functionally. As a result, the central lower back and left knee would gradually become the two worst pain sites. Besides the compensatory mechanism, ageing was another contributing factor. In Hong Kong, the construction industry is experiencing an ageing workforce. The ageing process predisposes workers to lower back pain which caused some disability [30]. If left unattended, the pain could become chronic and difficult to recover and turn into disability in their old age [31,32]. It is important to note that more than half of the construction workers in Hong Kong were employed on a daily basis which would make them more reluctant to forgo their daily wages to attend to their pain. They would continue working unless pain persisted to an intolerable level. The study showed that the daily activities of those workers with multiple pain sites and bilateral pain sites were interfered. The above observation brings forth the necessity of using health education or primary care to enhance early intervention.

Basically the relief methods used by the workers were self-help in nature, not relying on others and were comparatively cheap. The worrying fact was that only 37% of the time they found the pain relief methods effective, which implied that they often suffered from pains. Here, it is worthwhile to note that stretching exercises have been well documented in literature as a good method in the prevention and management of pain. Yet, exercising was one of the least pain relief methods used by the workers. Hence, it is advisable to conduct more health education at the workplace on self-help pain relief methods like stretching exercises, sleep enhancement, or mindfulness that are cost-effective.

## 5. Conclusions

Even though the point prevalence of the workers’ pain suffering was found to be mild at 10.6%, this study showed that the daily activities of those with multiple or bilateral pain sites had been interfered. Early intervention is the most cost effective way of handling pain. One way is to educate workers to take rest days to attend to their pain early. Another way is to organise health education and promotion on stretching exercises, sleep enhancement, or mindfulness to manage their pain. Pain when intervened early is reversible. Hopefully, these methods will save workers from pain deterioration and enhance their productivity.

## Figures and Tables

**Table 1:** Distribution of subjects’ demographic characteristics and occupational background by pain status (*n* = 2021).

Demographics		Frequency (%)		*p*-Value by X^2^
		Pain	Non-Pain	
Gender				0.723
	Female	36 (16.7)	320 (17.7)	
	Male	179 (83.3)	1486 (82.3)	
Age Group				0.306
	18–24	7 (3.3)	102 (5.7)	
	25–34	41 (19.1)	284 (15.7)	
	35–44	53 (24.7)	400 (22.2)	
	45–54	63 (29.3)	532 (29.5)	
	55–64	48 (22.3)	430 (23.8)	
	≥65	3 (1.4)	56 (3.1)	
Ethnicity				0.220
	Hong Kong	182 (84.7)	1532 (85.0)	
	Mainland China	31 (14.4)	234 (13.0)	
	Nepal	0 (0.0)	22 (1.2)	
	Pakistan	2 (0.9)	6 (0.3)	
	Others	0 (0.0)	9 (0.5)	
Highest Education Level Attained				0.652
	No Schooling	8 (3.7)	58 (3.2)	
	Primary School	39 (18.2)	385 (21.4)	
	Lower Secondary School	83 (38.8)	669 (37.1)	
	Upper Secondary School	43 (20.1)	344 (19.1)	
	Post-secondary (Non-degree)	18 (8.4)	136 (7.6)	
	Degree	5 (2.3)	61 (3.4)	
	Others	18 (8.4)	148 (8.2)	
Body Mass Index				0.983
	Underweight (<18.5)	4 (1.9)	42 (2.4)	
	Normal (18.5–22.9)	68 (32.7)	583 (32.8)	
	Overweight (23.0–24.9)	50 (24.0)	427 (24.0)	
	Obese (≥25.0)	86 (41.3)	728 (40.9)	

Note: The result was considered as statistically significant when *p* ≤ 0.05. Missing data of less than 2% for some variables due to non-responses.

**Table 2:** Distribution of trades by pain status (*n* = 2021).

Trade	Frequency (%)	
	Pain (*n* = 200)	Non-Pain (*n* = 1732)
General Labour	75 (37.5)	695 (40.1)
Electrical Fitter	17 (8.5)	90 (5.2)
Plant and Equipment Operator	17 (8.5)	146 (8.4)
Leveller	10 (5.0)	86 (5.0)
Air-conditioning Mechanic	8 (4.0)	18 (1.0)
Bar Bender and Fixer	8 (4.0)	52 (3.0)
Carpenter	8 (4.0)	61 (3.5)
Lift and Escalator Mechanic	7 (3.5)	109 (6.3)
Plumber	7 (3.5)	20 (1.2)
Cement Sand Mortar Worker	5 (2.5)	29 (1.7)
Piling Operator	5 (2.5)	35 (2.0)
General Welder	4 (2.0)	63 (3.6)
Electrical Wireman	4 (2.0)	20 (1.2)
Metal Worker	3 (1.5)	24 (1.4)
Painter and Decorator	3 (1.5)	37 (2.1)
Metal Scaffolder	0 (0.0)	21 (1.2)
Concretor	2 (1.0)	19 (1.1)
Mechanical Fitter	2 (1.0)	0 (0.0)
Plaster	2 (1.0)	6 (0.3)
Truck Driver	2 (1.0)	31 (1.8)
Rigger/Metal Formwork Erector	1 (0.5)	17 (1.0)

Note: Total n is not equal to 2021 due to the omission of some minor trades.

**Table 3:** Behavioural characteristics of workers by pain status (*n* = 2021).

Demographics		Frequency (%)		*p*-Value by X^2^
		Pain	Non-Pain	
Work Intensity				
	Light Work Intensity	35 (17.5)	305 (17.6)	0.631
	Moderate Work Intensity	161 (80.5)	1371 (79.2)	
	Heavy Work Intensity	4 (2.0)	56 (3.2)	
Wage Type				
	Daily	130 (60.5)	1080 (59.9)	0.880
	Monthly	85 (39.5)	722 (40.1)	
Experience in Construction (Year) ^#^		14.09 (11.30)	12.81 (11.14)	0.115
	Mean (Standard Deviation)			
Experience in Current Company (Year) ^#^		6.66 (8.36)	5.38 (7.47)	0.035 *
	Mean (Standard Deviation)			
Experience in Current Trade (Year) ^#^		11.51 (10.65)	10.67 (10.53)	0.274
	Mean (Standard Deviation)			
Working Hours/Day ^#^		8.93 (1.36)	9.00 (1.38)	0.465
	Mean (Standard Deviation)			
Working Days/Week ^#^		5.97 (0.46)	5.97 (0.38)	0.948
	Mean (Standard Deviation)			
Average Sleep Hours/Day ^#^		6.78 (1.17)	7.02 (1.09)	0.005 *
	Mean (Standard Deviation)			

Note: * The result was considered as statistically significant as *p* ≤ 0.05; ^#^ Analyzed by independent *t*-test; Missing data of less than 0.6% for some demographics due to non-responses.

**Table 4:** Pain intensities of workers (*n* = 215).

Pain Intensity	Mean	SD
Worst pain intensity in the last 24 h	3.17	1.969
Least pain intensity in the last 24 h	1.58	1.526
Average pain intensity in the last 24 h	2.31	1.553
Current pain intensity	1.70	1.445

Note: Pain intensity on a 0–10 scale. Missing data of less than 1% for some variables due to non-responses.

**Table 5:** Pain interference with workers (*n* = 215).

Domains of Life Activities	Pain Interference Score	
	Mean	SD
Daily Life	2.60	2.457
Mood	2.18	2.330
Walking	2.13	2.556
Work	2.78	7.049
Relationship with Others	1.20	2.054
Sleep	2.37	2.563
Enjoyment in Life	1.85	2.322

Note: Pain interference score on a 0–10 scale.

**Table 6:** Workers’ pain scores based on Brief Pain Inventory (BPI) (*n* = 215).

BPI Score	Mean	SD
Pain Severity Score (0–40)	8.78	5.054
Pain Interference Score (0–70)	14.68	13.358
Total Pain Score (0–110)	23.46	16.294

Note: Missing data of less than 1% for some variables due to non-responses.

**Table 7:** Distribution of workers’ pain scores by gender (*n* = 215).

BPI Score	Mean (SD)		*t*	*p*-Value
	Female	Male		
Pain severity Score (0–40)	10.25 (5.644)	8.49 (4.890)	−1.921	0.056
Pain Interference Score (0–70)	15.11 (13.821)	14.60 (13.301)	−0.211	0.833
Total Pain Score (0–110)	25.36 (17.886)	23.08 (15.978)	−0.765	0.445

Note: Missing data of less than 1% for some variables due to non-responses.

**Table 8:** Distribution of workers’ pain scores by age group (*n* = 213).

Age Group	Mean (SD)		
	Pain Severity Score (0–40)	Pain Interference Score (0–70)	Total Pain Score (0–110)
18–24	8.14 (2.035)	9.43 (7.786)	17.57 (9.307)
25–34	8.71 (4.232)	17.24 (14.221)	25.95 (16.942)
35–44	7.90 (5.439)	13.77 (10.989)	21.65 (14.302)
45–54	9.05 (4.607)	14.85 (14.417)	23.90 (16.940)
55–64	9.63 (6.115)	14.23 (14.341)	23.85 (17.994)
≥65	7.67 (3.767)	11.67 (13.868)	19.33 (14.012)
df	5, 207	5, 208	5, 207
F	0.663	0.605	0.550
*p*-value	0.652	0.696	0.738

**Table 9:** Differences in pain scores with multiple and bilateral pain sites (*n* = 215).

BPI Score	Mean (SD)		*t*	*p*-Value
	No Pain	Pain		
*Multiple Pain Sites*				
Pain Severity Score (0–40)	8.21 (5.301)	9.05 (5.028)	−1.053	0.294
Pain Interference Score (0–70)	11.35 (11.294)	15.85 (13.869)	−2.188	0.030 *
Total Pain Score (0–110)	19.56 (14.161)	24.90 (16.894)	−2.119	0.035 *
*Bilateral Pain Sites*				
Pain Severity Score (0–40)	8.39 (5.107)	9.83 (4.798)	−1.854	0.065
Pain Interference Score (0–70)	13.06 (13.042)	18.95 (13.347)	−2.934	0.004 *
Total Pain Score (0–110)	21.45 (15.873)	28.84 (16.318)	−3.003	0.003 *

Note: * The result was considered as statistically significant as *p* ≤ 0.05. Missing data of less than 4% for some variables due to non-responses.

**Table 10:** Differences in pain scores with or without 5 min of exercises before and after work.

BPI Score	Mean (SD)		*t*	*p*-Value
	Without	With		
*Five Minutes’ Exercise before work*				
Pain Severity Score (0–40)	8.56 (5.032)	9.00 (5.090)	−0.638	0.524
Pain Interference Score (0–70)	14.67 (13.662)	14.69 (13.127)	−0.010	0.992
Total Pain Score (0–110)	23.23 (16.984)	23.69 (15.683)	−0.204	0.838
*Five Minutes’ Exercise after work*				
Pain Severity Score (0–40)	8.93 (4.929)	8.39 (5.408)	0.694	0.488
Pain Interference Score (0–70)	14.96 (13.551)	13.95 (12.911)	0.489	0.625
Total Pain Score (0–110)	23.88 (16.626)	22.32 (15.434)	0.847	0.535

Note: Missing data of less than 1% for some variables due to non-responses.
